# Prophylactic Intrawound Vancomycin Powder in Minimally Invasive Spine Stabilization May Cause an Acute Inflammatory Response

**DOI:** 10.7759/cureus.28881

**Published:** 2022-09-07

**Authors:** Yuki Hyodo, Takeshi Arizono, Akihiko Inokuchi, Takahiro Hamada, Ryuta Imamura

**Affiliations:** 1 Department of Orthopaedic Surgery, Kyushu University Hospital, Fukuoka, JPN; 2 Department of Orthopaedic Surgery, Kyushu Central Hospital of the Mutual Aid Association of Public School Teachers, Fukuoka, JPN

**Keywords:** c-reactive protein, acute inflammatory response, surgical site infection, mrsa, vancomycin, instrumentation, lumbar spinal stenosis, minimally invasive posterior lumbar interbody fusion, minimally invasive spine stabilization

## Abstract

Introduction

Surgical site infections (SSIs) with methicillin-resistant *Staphylococcus aureus* are serious complications of spinal instrumentation surgery. Many spine surgeons are concerned that using prophylactic vancomycin powder will lead to certain risks: the development of multidrug-resistant pathogens, anaphylactic reactions, and organ toxicity. Minimally invasive spine stabilization (MISt) is associated with shorter operation times and less blood loss and may therefore require the use of less vancomycin powder, which may reduce these risks. This retrospective comparative study of patients who underwent MISt at a single institution aimed to evaluate the complications (such as allergy, SSIs, and organ toxicity) and the local and serum levels associated with using prophylactic intrawound vancomycin powder compared with IV cefazolin alone.

Methods

Thirty-four patients received intrawound vancomycin powder (1 g) applied during wound closure in minimally invasive posterior lumbar interbody fusion (MIS-PLIF). This group was compared with 133 control patients who did not receive vancomycin. White blood cell counts and C-reactive protein (CRP) levels were measured for both groups on postoperative days (PODs) 1, 3, and 7 and were statistically analyzed. In the vancomycin group, serum vancomycin levels were measured on PODs 1, 3, 7, and 14; drain vancomycin levels and postoperative blood loss were determined on PODs 1 and 2.

Results

The CRP levels on PODs 1 and 3 were significantly higher in the vancomycin group than in the control group (P<0.001, P=0.024). In the vancomycin group, mean drain levels trended downward from 313 μg/mL (POD 1) to 155 μg/mL (POD 2). These levels correlated negatively with drain drainage volume on both days (POD 1: r=-0.48, P=0.015; POD 2: r=-0.47, P=0.019). Mean serum vancomycin levels also trended downward from 2.3 μg/mL (POD 1) to 1.7 μg/mL (POD 14).

Conclusions

Our results unexpectedly demonstrated that the local application of vancomycin powder causes an acute inflammatory response and the long-term detection of low serum vancomycin levels. Less than 1 g of intrawound vancomycin powder may be useful only at high risk of SSI.

## Introduction

Surgical site infections (SSIs) are serious complications of spinal instrumentation surgery. In many cases, repeated surgical interventions and long-term antibiotic administration are necessary to control infections, which results in prolonged hospital stays, increased medical costs, reduced patient satisfaction, and poorer functional prognosis [1,2].

Guidelines in many countries recommend using intravenous (IV) cefazolin to prevent postoperative SSIs in spinal instrumentation surgery. However, the occurrence of difficult SSI cases with methicillin-resistant *Staphylococcus aureus* (MRSA) infections has been increasing [3]. The penetration of IV vancomycin into the spinal region is poor, and the in vivo vancomycin levels may often be lower than the minimum inhibitory concentration (MIC) of MRSA, which is greater than 1 μg/mL [4-7]. Also, IV vancomycin has been associated with hypotension, anaphylactic reactions, red man syndrome, inhibition of osteoblasts with resultant pseudoarthrosis, renal toxicity, selection and growth of gram-negative organisms, and development of vancomycin-resistant organisms in the oropharyngeal, respiratory, and genitourinary tracts [4,5,8]. On the other hand, previous studies showed that adjunctive local application of vancomycin powder reduced the number of postoperative infections in posterior instrumented thoracolumbar spinal fusions with relative safety compared to IV vancomycin [9,10].

Minimally invasive spine stabilization (MISt) is associated with shorter operation times and less blood loss [11]. We believe that less vancomycin powder used in MISt reduces SSI rates and lowers the risk of developing multidrug-resistant pathogens, anaphylactic reactions, and its related complications. The present study aimed to evaluate the complications (such as allergy, SSIs, and organ toxicity) and the local and serum levels associated with using prophylactic intrawound vancomycin powder compared with IV cefazolin alone in MISt.

## Materials and methods

This retrospective comparative cohort study included patients who underwent minimally invasive posterior lumbar interbody fusion (MIS-PLIF) at a single institution between April 2014 and June 2017. A total of 175 patients were initially identified. Eligible to be included in the study were patients who had a lumbar degenerative disease and were treated via minimally invasive procedures, via posterior lumbar interbody arthrodesis, including pedicle screws and interbody cages at one level with at least one year of follow-up. Exclusion criteria included infectious diseases, traumatic pathologies, and neoplastic and multi-level fusion cases. All operations were performed by two out of the three spine surgeons at our institution. There were no differences in surgical technique, implant, and protocol among surgeons. The study protocol was approved by the Institutional Review Board of the authors’ affiliated institution, Kyushu Central Hospital of the Mutual Aid Association of Public School Teachers (approval number 236). Patients were informed about the study design and methods and agreed to participate in this study.

All patients received standard antibiotic prophylaxis with 2 g of IV cefazolin within 1 hour of surgical incision followed by IV cefazolin every 8 hours for 48 hours. Patients in the vancomycin group also received 1 g of vancomycin powder directly into the local wounds during closure; approximately 0.5 g of vancomycin powder was applied to the midline incision, and the remaining 0.5 g was applied evenly to the right and left lateral incisions made for the percutaneous pedicle screws.

After surgery, we regularly assessed the patients’ body temperature and the presence or absence of complications (such as allergies, SSIs, and renal toxicity). We measured the number of white blood cells (WBCs) and the levels of C-reactive protein (CRP) on postoperative days (PODs) 1, 3, and 7. For the vancomycin group, we obtained blood samples from wound drains on PODs 1 and 2 to determine postoperative blood loss and vancomycin levels. We also measured serum vancomycin levels on PODs 1, 3, 7, and 14.

All statistical analyses were performed with EZR (Saitama Medical Center, Jichi Medical University, Saitama, Japan, version 1.54), which is a graphical user interface for R (The R Foundation for Statistical Computing, Vienna, Austria). We used Mann-Whitney U-tests to compare averages of continuous variables and chi-square tests to compare proportions of categorical variables between groups. We used the Spearman correlation coefficient to determine the relationship between drain drainage volume and vancomycin levels. Power analysis was performed using G*Power ver. 3.1.9.6 (freeware, Franz, Universitat Kiel, Germany). With a power of 80%, 0.05 level of statistical significance, and effect size of 1, the sample size for Mann-Whitney U-tests was calculated to be over 18. The significance threshold was P<0.05.

## Results

This study involved 167 patients who met the inclusion criteria. Thirty-four patients who were treated between April 2014 and June 2015 were included in the vancomycin group, and 133 patients who were treated between July 2015 and June 2017 were in the control group. Table 1 shows that the vancomycin group and the control group were statistically similar in terms of demographics.

**Table 1:** Patient Demographics Data (continuous covariates) are presented as median and range.

Characteristics	Vancomycin group (n=34)	Control group (n=133)	P-value
Age (years)	71 (25-84)	67 (14-89)	0.26
Sex (female), n (%)	18 (53)	53 (39)	0.17
Body mass index	24.8 (14.0-32.9)	24.3 (16.1-38.9)	0.93

Both groups had no complications such as allergy, renal toxicity, and SSIs within 30 days. The two groups did not show significant differences in WBC counts and CRP levels on POD 7 (Table 2).

**Table 2:** Laboratory Findings and Body Temperature POD, postoperative day Data (continuous covariates) are presented as median and range.

	Vancomycin group (n=34)	Control group (n=133)	P-value
White blood cells (/μL)			
POD 1	10000 (4300-15600)	9100 (4400-17800)	0.053
POD 3	8800 (5300-13120)	8100 (3100-15800)	0.33
POD 7	4950 (3700-8350)	5350 (3900-11700)	0.55
C-reactive protein (mg/dL)			
POD 1	4.01 (1.58-7.91)	1.10 (0.04-9.69)	<0.001
POD 3	9.14 (5.90-15.0)	7.68 (0.35-19.1)	0.024
POD 7	3.13 (1.02-10.6)	2.51 (0.51-14.2)	0.32
Body temperature			
No. of days to return to normal temperature	5 (1-13)	4 (1-18)	0.16

However, on PODs 1 and 3, the CRP levels in vancomycin group patients were significantly increased compared with those in control group patients (P<0.001, P=0.024) although the WBC counts showed no significant difference in the two groups. The time to return to normal body temperature was longer in the vancomycin group than in the control group, but this measure did not reach statistical significance (Table 2).

**Figure 1:**
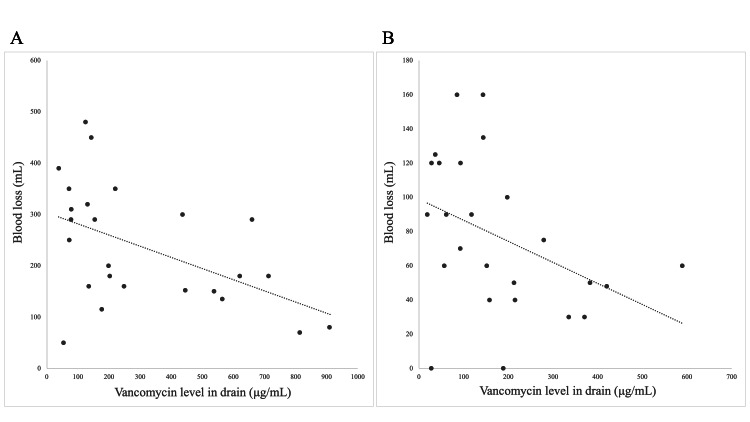
Drain Drainage Volume and Drain Vancomycin Levels Significant negative linear correlations were seen between drain drainage volume (postoperative blood loss) and drain vancomycin levels on postoperative days (PODs) 1 and 2. A: POD 1, r=-0.48, P=0.015. B: POD 2, r=-0.47, P=0.019

Mean vancomycin drug levels obtained from surgical drains in the vancomycin group peaked on POD 1 at 313 μg/mL (range 38-909) and dropped to 155 μg/mL (range 50-589) on POD 2. These levels correlated negatively with drain drainage volume (postoperative blood loss) on PODs 1 and 2 (Figure 1).

**Figure 2:**
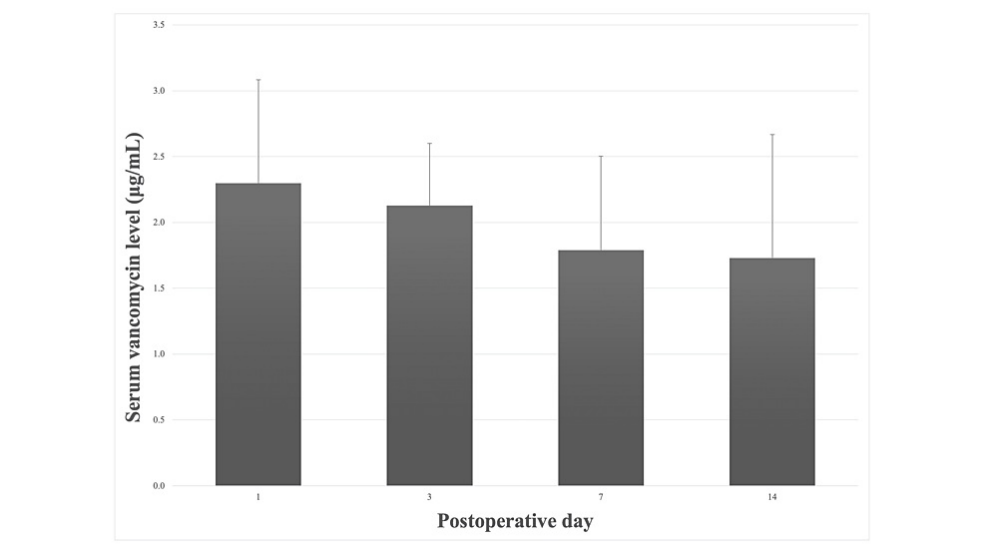
Serum Vancomycin Levels Mean serum vancomycin levels trended downward from postoperative days 1 to 14.

Figure 2 shows that mean serum vancomycin levels trended downward from 2.3 μg/mL (POD 1) to 2.1 μg/mL (POD 3) to 1.8 μg/mL (POD 7) to 1.7 μg/mL (POD 14).

## Discussion

This study determined that CRP levels in vancomycin group patients on PODs 1 and 3 were significantly increased compared with CRP levels in control group patients, but WBC counts and CRP levels after POD 7 were not significantly different in the two groups. We unexpectedly found that intrawound vancomycin powder caused an acute inflammatory response. In addition, local application of 1 g of vancomycin powder during MIS-PLIF achieved high vancomycin levels in surgical drains on PODs 1 and 2 and low serum vancomycin levels on PODs 1-14. These results suggest that high local vancomycin levels provide local antibiotic concentrations for at least two days postoperatively, and low serum vancomycin levels are unlikely to cause organ toxicity. On the other hand, low serum vancomycin levels had been unexpectedly detected for at least two weeks, and such long-term detection of vancomycin may lead to the development of vancomycin-resistant organisms.

MRSA has been reported as the most common causative microorganism isolated from SSIs [10,12,13]. SSIs after spine surgery are serious complications that negatively affect patient outcomes and greatly increase the morbidity and mortality of patients. Previous studies showed that intrawound application of vancomycin during spine surgery significantly reduced the risks of SSIs and returns to the operating room because of SSIs [9,10,14-19]. In addition, almost all current literature has reported no adverse outcomes attributable to the application of intrawound vancomycin powder [19]. Similarly, in our study, the vancomycin group did not have SSIs within 30 days and complications such as allergy and renal toxicity.

However, several case reports described occurrences of severe hypotension, anaphylactic reactions, and red man syndrome as a result of the local intrawound application of vancomycin powder as used for prophylaxis of infections [20-22]. These are allergic inflammations, and the erythrocyte sedimentation rate (ESR) and CRP are widely used as inflammatory markers. CRP was observed to have a higher diagnostic sensitivity and specificity than ESR in the diagnosis of various inflammatory conditions [23]. Suh et al. reported that on POD 3, the ESR in vancomycin group patients (25.2 ± 19.4 mm/hour) was significantly increased compared with that in control group patients (14.4 ± 14.2 mm/hour, P=0.004) [24]. In our study, the CRP levels on PODs 1 and 3 were significantly higher in the vancomycin group than in the control group. These findings suggest that intrawound vancomycin powder causes an acute inflammatory response including allergic inflammation and is not as safe as once believed.

Local application of vancomycin powder has produced wound drug levels higher than the MIC of MRSA, so infection rates may thereby be reduced. Sweet et al. reported that 2 g of intrawound vancomycin powder resulted in wound concentrations (128-1457 μg/mL) that were nearly 1000-fold higher than the MIC of MRSA in thoracic and lumbar posterior instrumented spinal fusions [10]. Armaghani et al. also reported that the application of 1 g of vancomycin powder in pediatric spinal deformity surgery produced local levels (115-403 μg/mL) that were well above the MICs of common pathogens [25]. As expected, our study here showed that the local concentration of vancomycin (155-313 μg/mL) was higher than the MIC of MRSA when 1 g of intrawound vancomycin powder was applied during MIS-PLIF. In addition, the intrawound application of vancomycin in spine surgery would be unlikely to impair bone healing and affect pseudoarthrosis rates [26-29]. In vitro studies have demonstrated that high local concentrations, up to 10,000 mg/mL, were necessary to inhibit osteogenesis. As demonstrated by our results, the observed local concentrations were well below this concentration.

Vancomycin powder was poorly absorbed from the wound site, so serum vancomycin levels may be much lower than the toxicity threshold (25 μg/mL) [25]. Sweet et al. reported that 80% of patients had undetectable vancomycin blood levels with a minimum sensitivity of 0.6 μg/mL, and only 20% had detectable vancomycin blood levels at low serum concentrations of 1.6 mg/mL on POD 1 [10]. Armaghani et al. reported that serum vancomycin levels trended downward from 2.5 μg/mL (POD 0) to 1.9 μg/mL (POD 1) to 1.1 μg/mL (POD 2) [25]. These low serum vancomycin levels are well below toxic levels (25 μg/mL) and are unlikely to cause organ toxicity. In addition, a meta-analysis indicated that topical vancomycin powder did not increase rates of gram-negative bacterial or polymicrobial spinal infections, as well as rates of infections with gram-positive bacteria, MRSA, and other microorganisms [30]. Our study, however, showed that mean serum vancomycin levels (1.7-2.3 μg/mL) were seen for at least two weeks after MIS-PLIF. This finding suggests that intrawound vancomycin powder was continuously absorbed, little by little, from the wound site. Such long-term detection of vancomycin may lead to some complications and reduced susceptibility or increased tolerance to vancomycin.

Spine surgeons have used empirical doses of 1-2 g of vancomycin powder; therefore, additional pharmacokinetic studies are needed to determine the optimal safe and effective dose for spine surgery [27]. A systematic review showed that patients undergoing MISt consistently had less blood loss than patients undergoing open transforaminal or posterior lumbar interbody fusion [11]. In our study, local vancomycin levels correlated negatively with postoperative blood loss and were much higher than the MIC of MRSA when 1 g of intrawound vancomycin powder was applied during MIS-PLIF; similar findings were noted for the use of 2 g in thoracic and lumbar posterior instrumented spinal fusions in adults [10] and 1 g in spinal deformity surgery in children [25]. For these reasons, less than 1 g of intrawound vancomycin powder during MIS-PLIF may be sufficiently effective to reduce the risk of SSIs.

Our study has certain limitations. First, we used CRP as a marker of an acute inflammatory response, but it is an imperfect measure of allergic inflammation and does not distinguish differences from other acute inflammatory responses. Second, we included various posterior surgical procedures for spinal conditions with multiple etiologies, and the CRP levels may vary depending on the etiology and operative procedure. Third, this is a retrospective study at a single center. The retrospective data review limited our ability to deduce causal relationships. The number of patients is too small to discuss the complications. Lastly, the possibility of a selection bias cannot be denied; however, we minimized this bias by dividing the two groups according to the time of surgery. A prospective comparative study of a larger number of patients may be necessary to achieve an accurate assessment of vancomycin use.

## Conclusions

We unexpectedly found that intrawound vancomycin powder induces an acute inflammatory response and the long-term detection of low serum vancomycin levels. The inflammatory response may be related to adverse events, including allergic inflammation, and the long-term detection of vancomycin may lead to the development of drug-resistant organisms. On the other hand, we identified no complications, and the local application of less than 1 g of vancomycin powder during MIS-PLIF may reduce the risk of SSIs. These findings suggest that the minimum dose of intrawound vancomycin powder is effective only in patients undergoing MIS-PLIF and at high risk of SSI. Additional studies are needed to determine the optimal dose of vancomycin powder, assess safety immediately after local vancomycin powder application, and evaluate the long-term safety and effectiveness of this drug treatment.
